# Modeling arterial pulse waves in healthy aging: a database for in silico evaluation of hemodynamics and pulse wave indexes

**DOI:** 10.1152/ajpheart.00218.2019

**Published:** 2019-08-23

**Authors:** Peter H. Charlton, Jorge Mariscal Harana, Samuel Vennin, Ye Li, Phil Chowienczyk, Jordi Alastruey

**Affiliations:** ^1^Department of Biomedical Engineering, School of Biomedical Engineering and Imaging Sciences, King’s College London, King’s Health Partners, London, United Kingdom; ^2^Department of Clinical Pharmacology, King’s College London, King’s Health Partners, London, United Kingdom; ^3^Institute of Personalized Medicine, Sechenov University, Moscow, Russia

**Keywords:** aging, arteries, blood flow, database of virtual subjects, pulse wave

## Abstract

The arterial pulse wave (PW) is a rich source of information on cardiovascular (CV) health. It is widely measured by both consumer and clinical devices. However, the physical determinants of the PW are not yet fully understood, and the development of PW analysis algorithms is limited by a lack of PW data sets containing reference CV measurements. Our aim was to create a database of PWs simulated by a computer to span a range of CV conditions, representative of a sample of healthy adults. The typical CV properties of 25–75 yr olds were identified through a literature review. These were used as inputs to a computational model to simulate PWs for subjects of each age decade. Pressure, flow velocity, luminal area, and photoplethysmographic PWs were simulated at common measurement sites, and PW indexes were extracted. The database, containing PWs from 4,374 virtual subjects, was verified by comparing the simulated PWs and derived indexes with corresponding in vivo data. Good agreement was observed, with well-reproduced age-related changes in hemodynamic parameters and PW morphology. The utility of the database was demonstrated through case studies providing novel hemodynamic insights, in silico assessment of PW algorithms, and pilot data to inform the design of clinical PW algorithm assessments. In conclusion, the publicly available PW database is a valuable resource for understanding CV determinants of PWs and for the development and preclinical assessment of PW analysis algorithms. It is particularly useful because the exact CV properties that generated each PW are known.

**NEW & NOTEWORTHY** First, a comprehensive literature review of changes in cardiovascular properties with age was performed. Second, an approach for simulating pulse waves (PWs) at different ages was designed and verified against in vivo data. Third, a PW database was created, and its utility was illustrated through three case studies investigating the determinants of PW indexes. Fourth, the database and tools for creating the database, analyzing PWs, and replicating the case studies are freely available.

## INTRODUCTION

The arterial pulse wave (PW) is used for physiological assessment in both clinical medicine and consumer devices. The PW contains a wealth of information on the cardiovascular (CV) system (4). It is influenced by the heart, with properties such as heart rate (HR) and stroke volume (SV) influencing its duration and morphology, and the vasculature, with arterial stiffness and wave reflections influencing its morphology. Consequently, a range of physiological parameters can be estimated from the PW, which are useful for diagnosis, monitoring, and clinical decision making. The PW can be easily measured using noninvasive clinical devices, such as oscillometric blood pressure (BP) monitors and pulse oximeters. It is also routinely monitored by consumer devices, such as smart watches and fitness wristbands (27). As a result, there is scope for obtaining great insight into CV function from the PW in clinical settings and daily life.

The PW has been the subject of much in vivo research. For instance, the physiological determinants of pulse wave velocity (PWV) and late systolic pressure augmentation have been investigated in both large observational studies (37, 127a) and smaller interventional studies (105, 168). In addition, techniques for estimating physiological parameters from PWs have been assessed in clinical studies, including estimating cardiac output (CO) from invasive pressure PWs (152), estimating arterial stiffness from noninvasive pressure PWs (68), and estimating an aortic pressure wave from a peripheral PW (116). While in vivo studies are valuable, they do have disadvantages, as described in Willemet et al. (170): it can be difficult to measure reference variables precisely (e.g., CO or arterial stiffness); it is difficult to study the influence of individual CV properties on the PW in vivo, since other properties may change concurrently; it can be difficult to measure PWs at all sites of interest (particularly central arteries); clinical trials are expensive and time-consuming; and in vivo measurements are subject to experimental error.

One-dimensional (1D) computational modeling provides a complementary approach for research into the PW, as it allows PWs to be simulated under different CV conditions (145). Indeed, in silico studies using computational modeling have been performed to complement the aforementioned clinical studies: the determinants of PWV and pressure augmentation were assessed in Willemet et al. (169, 170), and techniques for estimating CO, arterial stiffness, and the aortic pressure wave have been assessed (115, 150, 156). While there are also disadvantages to in silico studies (e.g., reliance on modeling hypotheses), they can provide additional hemodynamic insights, which would be difficult to obtain in vivo, and can be used for preliminary design and assessment of PW analysis techniques across a wide range of CV conditions in a relatively quick and inexpensive manner. Furthermore, the results of in silico studies can be used to inform the design of in vivo studies (170) and to confirm the findings of in vivo studies (89, 161).

The aim of this study is to develop and verify an approach for simulating PWs representative of a sample of healthy adults. Such an approach would be useful for in silico studies of hemodynamics and PW indexes, as the results could be indicative of those that would be obtained in vivo. The approach presented here combines novel methods with several recent developments in 1D modeling from the literature. The main goals were to *1*) develop methods for simulating PWs during healthy aging, exhibiting normal physiological variation; *2*) develop a method for simulating photoplethysmogram (PPG) PWs, which are widely measured by pulse oximeters and consumer devices; *3*) create a database of PWs, representative of a sample from a healthy adult population, and verify it through comparison with in vivo data; *4*) present case studies demonstrating the utility of the approach; and *5*) make the PW database and accompanying code freely available to support further research. This builds on preliminary work (23, 24, 31, 34).

## MATERIALS AND METHODS

### Modeling Arterial Pulse Waves

**Fig. 1.**
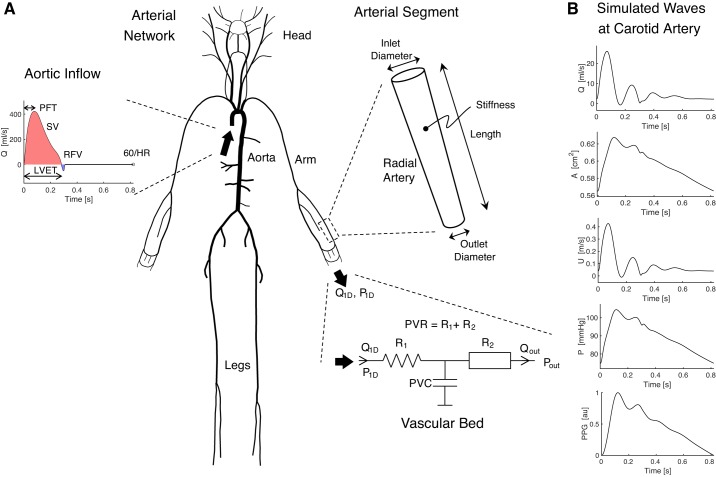
The one-dimensional model of pulse wave propagation (*A*) and simulated pulse waves (*B*). The model consists of an arterial network consisting of arterial segments making up the larger arteries, an aortic inflow waveform prescribed at the aortic root, and lumped boundary conditions at each terminal segment representing vascular beds. See glossary for definition of terms. [Adapted from Charlton et al. (30), licensed under CC-BY 3.0; https://creativecommons.org/licenses/by/3.0/.]

The 1D formulation of PW propagation was used to simulate arterial PWs numerically (107). The computational model was based on that described in Alastruey et al. (2). It consisted of three key components, as shown in Fig. 1. First, the arterial network was decomposed into 116 arterial segments making up the larger arteries of the thorax, limbs, and head. Arterial segments were modeled as thin, viscoelastic tubes of constant length and linearly tapered diameter (30). Second, a periodic inflow waveform was prescribed as a boundary condition at the aortic root, modeling flow from the left ventricle. Third, terminal three-element windkessel boundary conditions were imposed at the outlets of peripheral arterial segments, modeling vascular beds.

The nonlinear 1D equations of incompressible and axisymmetric flow in Voigt-type viscoelastic vessels were used to model blood flow, based on the physical principles of conservation of mass, momentum, and energy (30). Key assumptions were as follows: laminar flow, incompressible and Newtonian blood (density, ρ = 1,060 kg/m^3^, and viscosity, μ = 2.5 mPa·s), parabolic flow, and no energy losses at bifurcations. The previously described model provided four types of arterial PWs: blood flow velocity (*U*), luminal area (*A*), volume flow rate (*Q *=* UA*), and BP (P) waves. In this study, the model was extended to simulate PPG PWs by assuming that the PPG is dependent on the volume of arterial blood in a tissue. At the periphery, the PPG PW was calculated from the volume of blood stored in the terminal windkessel model. Within the arterial network, the PPG was calculated from the volume of blood stored in the arterial segment. In both cases, the PPG was calculated by normalizing the pulsatile variation in blood volume to occupy a range of 0 to 1.

For further details of the model, including the geometry of the arterial network and the methodology for simulating PPG PWs, see the appendix, *Numerical Model*.

### Prescribing Model Input Parameters for Different Ages Based on a Literature Review

**Fig. 2.**
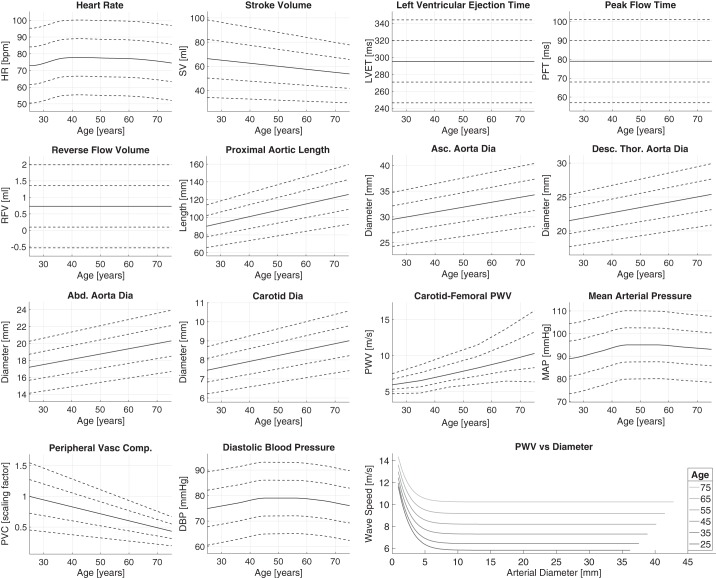
A summary of the literature review findings. The mean (solid line) and SD (dashed lines indicating ±1 and ±2 SD) values are shown for each parameter. The positive and negative SD values for carotid-femoral pulse wave velocities (PWVs) are different to capture the positive skewness of this variable’s distribution. The final wave speed plot shows the baseline wave speed as a function of diameter for each age. See glossary for definition of terms.

The model input parameters were adjusted to simulate PWs representative of healthy adults at each age decade from 25 to 75 yr. The parameters can be categorized as follows: cardiac, arterial, vascular bed, and blood properties. Referring to Fig. 1, the cardiac properties influence the aortic inflow waveform; the arterial properties determine the mechanical and geometrical characteristics of arterial segments; and the vascular bed properties are captured by the components of the vascular bed model. In this section, we present a review of the literature describing changes in these properties with age, including findings from 97 articles, and describe the methods used to extract values for the mean and intersubject variation of each model input parameter at each age decade. The findings for each parameter are presented in the appendix, *Literature Review*. The most reliable studies reporting the mean and intersubject variation of each parameter at each age were identified using the following criteria: *1*) whether the reported change with age was in keeping with the consensus from the review; *2*) the accuracy of the technique used to measure the parameter; and *3*) the nature of the subjects studied (namely their level of health, age range, and sample size). The typical values found for a sample of healthy adults are shown for each parameter in Fig. 2, and the equations describing them as a function of age are provided in the appendix, *Literature Review*.

#### Cardiac properties.

Cardiac properties were specified to the model through an inflow waveform prescribed at the aortic root (shown in Fig. 1). The waveform is affected by the following: HR, SV, left ventricular ejection time (LVET), peak flow time (PFT), reverse flow volume (RFV), and aortic flow waveform morphology. These characteristics are now considered in turn.

The vast majority of the identified articles that investigated changes in HR with age (7, 15, 36, 45, 54, 69, 82, 95, 102, 103, 108, 121, 124, 127, 128, 131, 132, 136, 141, 147, 173, 174) did not find a change with age (see Table A1). Yashin et al. (173) reported a nonlinear change in HR between the ages of 28 and 90 yr in Framingham Heart Study data (*n* = 5,209): an increase until around 55 yr in men, followed by a slight decrease until age 70 yr, and a rapid decline thereafter. The change observed in this study was small, with the mean HR varying between 67 and 76 beats/min for men. When combined with the nonlinear nature of the change, and the inclusion of older subjects in this study, this may explain why most other studies did not identify a change. This study was used to model changes in HR with age since it was population based and far larger than the others. Mean values for each age were obtained by interpolating the male data from this study using shape-preserving piecewise cubic interpolation. Values for normal variation in HR were not provided by this paper. Therefore, a standard deviation of 11 beats/min was obtained from a population study of 800 UK Biobank participants, aged 45–74 yr old (118). It was assumed that this value remained constant with age. The HR was prescribed to the model by setting the duration of the inflow waveform, *T* = 60/HR.

The majority of the identified articles (20, 22, 69, 88, 102, 108, 118, 120, 121, 131, 132) indicated that SV decreases with age. The largest study was an analysis of echocardiographic data acquired from 3,719 subjects (120). This study was chosen to model both the change in SV with age and normal variation in SV. The mean and standard deviation values for SV at each age were estimated from the upper and lower male reference values by assuming a normal distribution. SV was input to the model by setting the integral of the input flow waveform, *Q*(*t*), as $\overset{T}{\underset{0}{\int }}Q\left(t\right)\text{d}t=\text{SV}$, where *t* is time and *T* is the duration of a cardiac cycle.

The majority of the identified studies (54, 55, 67, 103, 121, 124, 136, 144, 154, 171) observed no change in LVET with age. Gold standard measurement techniques (echocardiograms and Doppler aortic flow signals) were used in three studies with low numbers of subjects (83, 65, and 62 subjects), which all found no change in LVET with age (54, 55, 136). Other studies included data from over 350 subjects, but did not use gold standard measurements, instead using the duration of the systolic portion of the carotid flow or pressure signal (67, 171), the QT interval (103, 154), or phonocardiogram measurements (144). They reported a range of conclusions: no change (103, 121, 144), an increase (154), or a small nonlinear change (171). Therefore, it was assumed that LVET did not change with age. A mean value of 282 ms was obtained from Mynard and Smolich (107). Although this is slightly lower than the values of 295 ± 24 and 306 ± 22 ms reported in Gerstenblith et al. (55) and Salvi et al. (136), it was chosen because it provided more realistic PW shapes. Gerstenblith et al. (55) was used to model normal variation in LVET. Several articles have reported that LVET changes with HR (49, 64, 123, 135, 137, 144, 165, 166, 171) and SV (64, 123, 165). Data on the relationship between LVET, HR, and SV were reported in Weissler et al. (166). The data from normal subjects were used to calculate an empirical relationship

(1) LVET[ms]=244−0.926HR[beats/min]+1.08SV[ml]

which was used to model the changes in LVET with HR and SV.

There is little information in the literature on how the PFT is affected by age. A study of 82 healthy subjects, aged 21–78 yr, found no significant change in PFT with age when measured with gold standard aortic Doppler flow (54). Similarly, a study of 96 healthy subjects aged 19–79 yr also found no significant change (MRI measurements at ascending aorta) (15). In contrast, a study of PFT estimated from carotid pressure waves in 56 healthy subjects found a substantial decrease with age (67). Due to the limited and conflicting evidence, it was assumed that PFT did not change with age. A normal value of 79 ± 11 ms was obtained from echocardiography data in Kamimura et al. (73).

The ascending aortic flow waveform typically consists of a positive systolic flow wave, followed by a period of reverse flow (110). There is little information in the literature on RFV. Bensalah et al. (15) found no significant difference in RFV between young and elderly subjects in the ascending aorta (although they did observe an increase in peak backward *Q* with age). Similarly, Svedlund et al. (153) found no difference between the ratios of systolic to diastolic velocity time integrals in the aortic arch between younger and older subjects. Therefore, it was assumed that RFV did not change with age. A normal value of 0.73 ± 0.63 ml was obtained from ascending aortic data from Bensalah et al. (15).

The aspects of the aortic inflow waveform considered so far can be used to specify the integral of the waveform, its duration, and the timings of peak flow and end-systole. There is little evidence in the literature on how the remaining aortic flow wave characteristics vary with age and within age groups. Examples of aortic flow waveforms for young and old subjects are provided (15, 108, 110), although these are based on measurements from individual subjects. Therefore, it was assumed that the remaining aortic flow wave characteristics did not change with age or exhibit any variation. The morphology was modeled on the wave provided in Mynard and Smolich (107), since this has been previously shown to give reasonable PW simulations. Details of the methodology used to prescribe an inflow waveform with the desired characteristics are provided in the appendix, *The aortic inflow waveform*.

#### Arterial properties.

The following properties of arterial segments were specified to the model: length, inlet and outlet diameters, wall stiffness, and wall viscosity. These are now considered in turn.

Few studies have investigated how arterial lengths change with age. The length of the proximal aorta has been found to increase with age (15, 40, 66, 151). In contrast, the lengths of more distal sections of the aorta (42, 66, 151) and the carotid (151) and iliac (151) arteries have been found to either not change with age, or exhibit a complex change (in one case). Therefore, it was assumed that the proximal aorta (up to and including the aortic arch) lengthens with age, whereas the lengths of other arteries do not change. Baseline lengths for the 25 yr old were adapted from those in Alastruey et al. (3) and Mynard and Smolich (107). Relative changes in proximal aortic length with age were modeled using data from Hickson et al. (66), since it used reliable methodology (MRI measurements of the aortic arch, 157 subjects, aged 18–77 yr). However, it did not provide age-specific values for the normal variation in length. Therefore, normal variation was modeled using data from Bensalah et al. (15).

Several studies have investigated how the diameters of the aorta [ascending (1, 15, 21, 66, 81, 96, 102, 108, 126, 130, 157, 162, 163), descending thoracic (1, 66, 126, 130, 162), abdominal (66, 72, 117, 130, 149, 162)] and carotid artery (13, 16, 63, 67, 128, 139) change with age, with the vast majority indicating that both increase with age. In contrast, few studies investigated changes in the diameters of the iliac (72, 117), femoral (13, 138, 139), brachial (57, 160), or radial (16) arteries, and these reported a range of conclusions. Therefore, it was assumed that the diameters of the aorta and carotid artery increase with age, whereas the diameters of the remaining arteries are not affected by age. Baseline diameters for the 25 yr old were adapted from Alastruey et al. (3) and Mynard and Smolich (107). A study by Hickson et al. (66) (*n* = 157) was used to model changes in aortic diameter with age, since it contained data from all three aortic sites, from subjects free of CV disease and medication, over a wide age range (24–73 yr), acquired using MRI. However, this study did not provide data on normal variation in aortic diameter. Therefore, normal variation was modeled using data from Agmon et al. (1). Changes with age and normal variation in carotid artery diameter were modeled using data from Hansen et al. (63), since it used echo-tracking measurements from healthy subjects with a wide age range. The arterial diameters were prescribed at male age-specific diastolic BP (DBP) values from McEniery et al. (99), a study of 4,001 healthy subjects.

The literature on changes in PWV with age was reviewed to identify target PWVs for optimizing the stiffness of arterial segments. Many studies have investigated how PWV changes with age in the aorta (9, 10, 12, 15, 56, 62, 65, 66, 80, 91, 99, 102, 106, 111, 127, 127a, 142, 146, 159) and the arteries of the arms (9, 10, 18, 19, 50, 62, 65, 86, 99, 106, 148) and legs (9, 10, 43, 65, 91). The vast majority observed an increase in PWV with age. The largest study reported reference values of carotid-femoral PWV (*n* = 11,092) according to age and BP (127a). The subjects in this study were from eight European countries, free from overt CV disease, and aged from 15 to 97 yr. Therefore, this study was used to model changes in aortic PWV with age and mean arterial pressure (MAP). We found no similar population-level studies reporting how PWVs at the arm and leg change with age. Instead, relationships between aortic and brachial-radial (arm) and femoral-dorsalis pedis (leg) PWVs were calculated from the data in Avolio et al. (9) (*n* = 524). These relationships were then used to calculate desired values for arm and leg PWVs, corresponding to the desired aortic PWVs. Following studies (107, 113, 169), the stiffness of each segment was assumed to be related to its diastolic radius, *R*_d_, using

(2) $$Eh=R_{\text{d}}\left[k_{1}exp\left(k_{2}R_{\text{d}}\right)+k_{3}\right]$$

where *E* is the Young's modulus, *h* the wall thickness, and *k*_1_, *k*_2_, and *k*_3_ are empirical constants, which were optimized to provide theoretical wave speeds, *c*_d_, in keeping with the desired PWVs (for further details, see the appendix, *Arterial stiffness*). The *c*_d_ was calculated as (2)

(3) $$c_{\text{d}}=\sqrt{\frac{2Eh}{3\rho R_{\text{d}}}}$$

Wall viscosity, Γ, was calculated following Mynard and Smolich (107) as

(4) $$\Gamma =\frac{b_{1}}{2R_{\text{d}}}+b_{0}$$

where *b*_1_ = 150 g·cm·s^−1^ and *b*_0_ = 600 g/s are empirical constants, chosen to achieve realistic hysteresis in pressure-area curves at peripheral arteries. Wall viscosity was assumed to remain constant with age, as there is little evidence to suggest otherwise (76).

#### Vascular bed properties.

It is difficult to assess the properties of vascular beds in vivo. Therefore, we considered changes in systemic vascular properties reported in the literature and used these to inform the expected changes in vascular bed properties.

The majority of articles describing variations in systemic vascular resistance (SVR) with age (36, 45, 69, 75, 100, 102, 108, 125, 132) reported an increase with age. However, the two articles with the largest study cohorts (*n* = 623 and 200) found no change in SVR index (i.e., indexed to body surface area) and SVR in men (45, 125). Consequently, it was not clear whether SVR changes with age. Therefore, we calculated peripheral vascular resistance values which would result in realistic MAP values. Changes in MAP with age, and normal variation in MAP, were modeled using male data from McEniery et al. (99), the same study used for DBP. Mean values for each age were obtained by interpolating the data using cubic spline interpolation, whereas values for normal variation in MAP were obtained using linear interpolation. The resistance of each vascular bed was adjusted from its baseline value [specified in Mynard and Smolich (107)] to achieve the desired MAP. The total values for each bed were split between each branch feeding into that bed by setting the windkessel resistances to be inversely proportional to the branch’s luminal *A* (30).

All of the articles identified that investigated changes in systemic vascular compliance with age (35, 91, 93, 100, 129) reported a decrease with age. The largest studies estimated large- and small-artery compliances from brachial and radial pressure PWs (100, 129). These observed a reduction in both large- and small-artery compliances with age, indicating that the reduction in systemic vascular compliance with age is not solely caused by changes in larger arteries, but is also contributed to by the rest of the circulation. Therefore, it was assumed that peripheral vascular compliance (PVC) decreased with age. Baseline PVC values corresponding to the 25-yr-old model were obtained from Mynard and Smolich (107). Changes in PVC with age were modeled using the equation for oscillatory (small artery) compliance provided in McVeigh et al. (100). Normal variation in PVC was modeled using the results for oscillatory compliance reported in Resnick et al. (129).

#### Blood properties.

Blood density and viscosity were assumed to be constant, since there is little evidence to suggest they change with age (79).

### Generating a Database of Arterial Pulse Waves

**Fig. 3.**
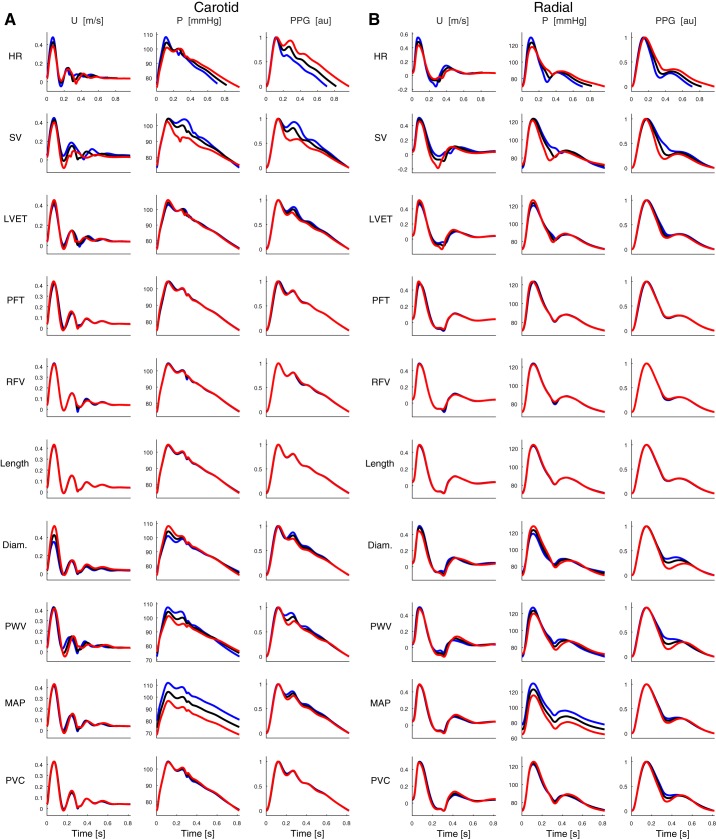
Pulse waves (PWs) for the 25-yr-old subject at the carotid artery (*A*) and the radial artery (*B*). The waves shown are at baseline (black), and those obtained when increasing (blue) and decreasing (red) each parameter independently by 1 SD from its baseline value. See glossary for definition of terms.

A preliminary set of PWs was created for the 25-yr-old subject to determine which CV properties should be varied in the database. PWs were first simulated using the baseline CV properties and then by changing each property independently by ±1 standard deviation (SD) from its mean value. The resulting PWs at the carotid and radial arteries are shown in Fig. 3. Six of the ten properties were found to strongly influence PWs (HR, SV, LVET, diameter, PWV, and MAP), whereas the remainder did not (PFT, RFV, proximal aortic length, and PVC). Only those properties that strongly influenced PWs were varied at each age to mimic normal physiological variation in the database.

A database of PWs was created by simulating PWs for subjects of each age decade from 25 to 75 yr. PWs were sampled at 500 Hz. First, PWs were simulated for a baseline subject at each age (using the age-specific mean value for all properties described in *Prescribing Model Input Parameters for Different Ages Based on a Literature Review* above). Second, PWs were simulated for 3^6^ = 729 subjects at each age by changing the six identified CV properties in combination with each other by ±1 SD from their age-specific mean values. This resulted in 6 × 729 = 4,374 subjects in the database. Third, the plausibility of each subject was investigated by comparing their aortic and brachial BPs [systolic BP (SBP), DBP, MAP, pulse pressure (PP), and PP amplification (PP_amp_)] to reference healthy values from McEniery et al. (99). A subject was deemed to exhibit implausible BPs if any of the BP measurements were outside 99% confidence intervals calculated as the age-specific mean ± 2.575 SD.

### Extracting Pulse Wave Indexes

PW indexes, which are commonly measured in clinical practice or research, were extracted from PWs. First, hemodynamic parameters were extracted from flow and pressure PWs at the aortic root. SV, CO, LVET, PFT, and RFV were extracted from the flow PW. HR and maximal dP/d*t* were extracted from the pressure PW. Second, SBP, DBP, MAP, and PP values were extracted from pressure PWs at common measurement sites. Third, PP_amp_ was calculated as the ratio of brachial to aortic PP. Fourth, pulse transit times (PTTs) were measured along the following paths: carotid-femoral, carotid-radial, femoral-ankle, aortic (i.e., aortic root to iliac bifurcation), and between the aortic root and each measurement site. PTTs were measured from pressure waves using the foot-to-foot algorithm (51, 53). PWVs were calculated from the PTTs and corresponding arterial path lengths. Fifth, indexes of arterial stiffness were calculated from the aortic root pressure PW (augmentation pressure (AP) and index (AIx), and the time to reflected wave) and the digital PPG [modified aging index (AGI_mod_), reflection index (RI), and stiffness index (SI)].

**Fig. 4.**
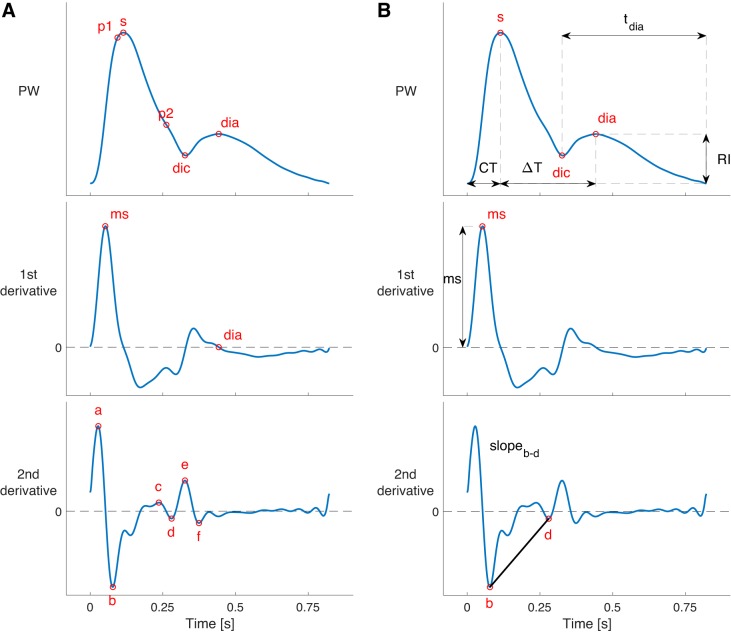
Pulse wave (PW) analysis, illustrated for a radial pressure PW. *A*: fiducial points were identified on the PW, and its first and second derivatives. *B*: several pulse wave indexes were calculated from the amplitudes and timings of these fiducial points, including those shown. See glossary for definition of terms.

A range of additional PW indexes that have been proposed in the literature were also calculated. The timings and amplitudes of the following fiducial points were calculated: P1, P2, systolic peak, and point of maximal dP/d*t* on the pressure PWs; a, b, c, d, e, systolic peak, diastolic peak, dicrotic notch, and point of maximal dPPG/d*t* on the PPG PWs. These points were identified using the *PulseAnalyse* script (described in the appendix, *Pulse Wave Analysis Algorithms*), which analyses the PWs and their derivatives, as shown in Fig. 4. P1 and P2 have previously been reported as the first inflection point and second systolic peak on the central pressure PW, indicative of the times of maximum aortic *U* and maximum AP due to wave reflection, respectively (90). They are used to calculate the AIx, as P1 occurs at the arrival of a reflected wave, and P2 occurs as the peak of the reflected wave. In addition, the following values were calculated at the aortic root: the volume of flow up to each of the times of P1 and P2, and *U* at P1 and P2. Finally, the mean, maximum, and minimum values of the *Q*, *U*, and *A* PWs were extracted.

### Comparison with In Vivo Data

The PW database was verified by comparing the simulated PWs with two sets of in vivo data from healthy subjects. First, the shapes of simulated PWs for virtual subjects of different ages were compared with in vivo PWs at different ages obtained from Flück et al. (46); normotensive subjects during screening for hypertension (including aortic root pressure PWs estimated using a transfer function) (89); and the Vortal data set (28, 29). Additional comparisons of PW shapes were performed using data from studies (5, 6, 41, 48, 67, 78, 100, 101, 172) (results not shown). Second, the hemodynamic characteristics of the simulated PWs were compared with the in vivo hemodynamic values reported in McEniery et al. (99).

### Case Studies

The utility of this approach for simulating PWs is demonstrated in three case studies. In the first study, we investigated the determinants of changes in PP_amp_ with age. To do so, we assessed the effects of age on early systolic amplification and late systolic aortic pressure augmentation, quantified as PP_amp_, calculated using the aortic PP at P1 and P2, respectively. Second, we investigated how well the following finger PPG PW indexes correlate with aortic PWV: RI (38); SI (104); and AGI_mod_ (158). Reference aortic PWV was calculated from pressure PWs using the foot-to-foot method (53), correlations were assessed using the coefficient of determination (*R*^2^, the square of the Pearson correlation coefficient), and the determinants of the indexes were assessed using the relative sensitivity index [which indicates the percent change in a PW index associated with a change in model input parameter of 1 SD from baseline (169)]. In the third study, we assessed how well algorithms for tracking CO perform during changes in CO and MAP from baseline. Two algorithms were implemented to estimate CO from the radial pressure PW based on the two-element windkessel model of the circulation (25). The first algorithm is based on the assumption that CO is proportional to the root mean square (RMS) of the radial pressure PW (25). The second algorithm is based on the assumption that CO is proportional to the ratio of PP to compliance, approximated as PP/[*T* × (SBP + DBP)], where *T* is the PW duration, and compliance is assumed to be proportional to mean BP (92, 115). These algorithms were chosen as it has been reported that similar algorithms are used in commercial devices (175). The algorithms were calibrated using the age-specific baseline simulations. Performance was assessed using the mean absolute percentage errors (MAPEs) of estimated COs in simulations in which either CO (i.e., HR or SV) or MAP were varied, while all other parameters were held at baseline.

## RESULTS

### Database Characteristics

**Fig. 5.**
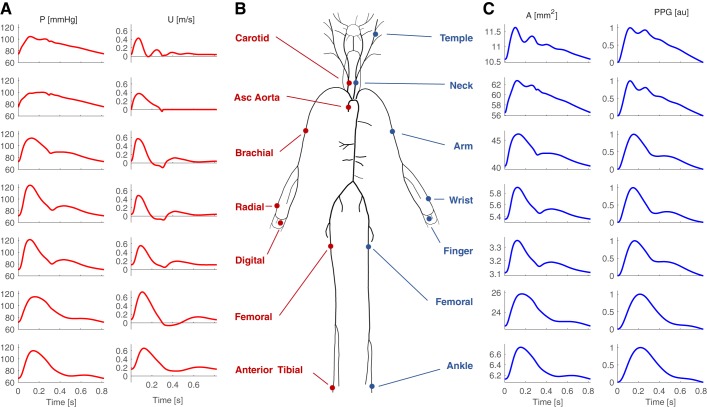
The pressure (P) and flow velocity (*U*) (*A*), luminal area (*A*) and photoplethysmogram (PPG) pulse waves (*C*) simulated at common measurements sites for the baseline 25-yr-old subject (*B*). [Adapted from Charlton et al. (30), licensed under CC-BY 3.0; https://creativecommons.org/licenses/by/3.0/.]

The PWs contained within the database are illustrated in Fig. 5. There are marked differences between PWs at different sites, such as the increase in systolic pressure and the transition from an A- to C-type pressure wave shape with distance from the aortic root (108); the genesis of a diastolic peak in *U* in the limbs, which is accompanied by diastolic peaks in the other PWs at limb sites; and the genesis of a second systolic peak in *U* at the carotid artery, accompanied by second systolic peaks in *A* and PPG PWs at the temporal artery, which bifurcates from the carotid artery.

A total of 537 out of the 4,374 virtual subjects exhibited BPs outside of healthy ranges. This was predominantly due to abnormal PP (observed in 431 subjects) and abnormally high PP_amp_ (90 of the remainder). Most of the subjects with abnormally high PP had increased PWV and often had at least one of increased SV, increased MAP, and decreased large-artery diameter. The subjects with abnormally low PP had the opposite characteristics: decreased PWV and at least one of decreased SV, decreased MAP, and increased diameter. Most of the remaining subjects with abnormally high PP_amp_ had decreased PWV and often increased HR or decreased MAP. The proportion of subjects exhibiting implausible BPs increased with age (from 3% of 25 yr olds to 32% of 75 yr olds). Only those subjects with BPs within healthy ranges were included in the following analyses.

The hemodynamic characteristics of the PW database are summarized in Table 1, showing the wide range of CV physiology exhibited by subjects in the database, both across the whole age range and at each age. Some of the parameters were prescribed to the model and were therefore predetermined, such as HR and proximal aortic length. In contrast, many of the hemodynamic PW parameters were not prescribed directly, but were determined from simulated PWs, such as SBP, PP_amp_, and carotid AIx. There were marked changes in these resultant parameters with age, indicating that the different values of input parameters prescribed at each age did result in changes in PW shape, as seen in vivo.

**Table 1.** The hemodynamic characteristics of the PW database for all physiologically plausible virtual subjects (n = 3,837) and for the subjects at each age, from 25 to 75 yr old

Hemodynamic Characteristic	All Subjects	25 yr	35 yr	45 yr	55 yr	65 yr	75 yr
Physiologically plausible subjects, *n*	3,837	712	684	654	641	588	558
*Cardiac*							
Heart rate, beats/min	75.6 ± 9.2	73.0 ± 9.1	76.3 ± 9.1	77.0 ± 9.0	77.0 ± 9.1	76.3 ± 9.0	74.4 ± 9.0
Stroke volume, ml	60.4 ± 12.4	66.8 ± 13.1	64.1 ± 12.5	61.3 ± 11.6	58.7 ± 11.1	55.8 ± 10.4	53.6 ± 9.8
Cardiac output, l/min	4.57 ± 1.09	4.88 ± 1.13	4.90 ± 1.13	4.72 ± 1.06	4.52 ± 1.02	4.25 ± 0.95	3.99 ± 0.86
Left ventricular ejection time, ms	283 ± 23	283 ± 23	284 ± 23	283 ± 23	282 ± 23	282 ± 23	282 ± 23
dP/d*t*, maximum aortic value, mmHg/s	573 ± 127	585 ± 130	572 ± 132	573 ± 126	570 ± 128	568 ± 119	568 ± 122
Peak flow time, ms	80.0 ± 0.2	79.9 ± 0.4	80.0 ± 0.0	80.0 ± 0.0	80.0 ± 0.0	80.0 ± 0.1	80.0 ± 0.2
Reverse flow volume, ml	0.7 ± 0.0	0.7 ± 0.0	0.7 ± 0.0	0.7 ± 0.0	0.7 ± 0.0	0.8 ± 0.1	0.8 ± 0.1
*Arterial*							
Aortic pressure, mmHg							
SBP	108.8 ± 10.1	100.1 ± 8.0	104.6 ± 8.4	110.1 ± 8.4	111.9 ± 8.7	113.6 ± 8.7	115.1 ± 9.4
DBP	75.9 ± 6.7	74.7 ± 5.7	77.3 ± 6.0	78.9 ± 6.1	77.4 ± 6.2	74.8 ± 6.6	71.7 ± 7.2
MAP	93.9 ± 6.5	89.2 ± 6.2	92.8 ± 6.1	96.3 ± 6.1	96.2 ± 6.0	95.4 ± 5.9	94.2 ± 5.8
PP	32.9 ± 11.1	25.4 ± 7.0	27.3 ± 8.3	31.3 ± 8.5	34.5 ± 9.4	38.9 ± 10.2	43.4 ± 12.3
Brachial pressure, mmHg							
SBP	118.1 ± 9.2	112.3 ± 8.7	115.9 ± 9.1	120.4 ± 8.6	120.6 ± 8.5	120.2 ± 8.3	120.1 ± 8.7
DBP	73.4 ± 6.7	72.0 ± 5.6	74.5 ± 6.1	76.3 ± 6.2	75.0 ± 6.3	72.3 ± 6.6	69.5 ± 7.1
MAP	93.7 ± 6.6	88.9 ± 6.1	92.5 ± 6.3	96.1 ± 6.1	96.1 ± 6.0	95.2 ± 5.9	94.0 ± 5.8
PP	44.7 ± 10.2	40.3 ± 8.2	41.5 ± 9.2	44.1 ± 9.1	45.6 ± 9.6	47.9 ± 9.8	50.6 ± 11.5
Pulse pressure amplification (ratio)	1.41 ± 0.21	1.62 ± 0.15	1.56 ± 0.16	1.44 ± 0.13	1.35 ± 0.13	1.26 ± 0.11	1.19 ± 0.10
Augmentation pressure (carotid), mmHg	8.0 ± 8.2	0.6 ± 3.0	2.5 ± 3.6	5.9 ± 4.4	9.4 ± 5.2	13.9 ± 6.4	18.8 ± 8.4
Augmentation index (carotid), %	20.6 ± 16.8	2.3 ± 10.4	8.4 ± 10.7	17.8 ± 10.2	25.9 ± 9.4	34.3 ± 8.9	41.5 ± 9.1
Time to reflected wave (carotid), ms	102.3 ± 19.3	122.4 ± 9.1	115.6 ± 11.7	104.7 ± 13.0	96.2 ± 13.9	87.2 ± 12.9	80.2 ± 13.2
Pulse wave velocity, m/s							
Aortic	7.6 ± 1.7	5.9 ± 0.6	6.5 ± 0.8	7.3 ± 0.9	8.0 ± 1.1	8.9 ± 1.3	9.7 ± 1.6
Carotid-femoral	8.1 ± 1.8	6.3 ± 0.7	6.9 ± 0.9	7.8 ± 0.9	8.5 ± 1.1	9.5 ± 1.4	10.4 ± 1.9
Brachial-radial	10.7 ± 1.7	8.9 ± 0.6	9.5 ± 0.8	10.4 ± 0.8	11.1 ± 1.0	12.0 ± 1.3	12.8 ± 1.6
Femoral-ankle	10.3 ± 1.7	8.7 ± 0.9	9.2 ± 1.1	10.1 ± 0.8	10.7 ± 1.0	11.6 ± 1.2	12.4 ± 1.5
Diameter, mm							
Ascending aorta	39.4 ± 3.5	36.7 ± 2.6	37.8 ± 2.7	39.0 ± 2.8	40.2 ± 2.9	41.4 ± 3.0	42.6 ± 3.0
Descending thoracic aorta	26.3 ± 2.3	24.4 ± 1.7	25.2 ± 1.8	26.0 ± 1.9	26.8 ± 1.9	27.6 ± 2.0	28.3 ± 2.0
Abdominal aorta	15.6 ± 1.3	14.5 ± 1.0	15.0 ± 1.1	15.4 ± 1.1	15.9 ± 1.1	16.3 ± 1.2	16.8 ± 1.2
Length of proximal aorta, mm	95.1 ± 10.9	80.0 ± 0.0	86.4 ± 0.0	92.8 ± 0.0	99.2 ± 0.0	105.6 ± 0.0	112.0 ± 0.0
Modified aging index, au	−0.78 ± 0.46	−0.98 ± 0.24	−1.00 ± 0.25	−0.89 ± 0.33	−0.76 ± 0.43	−0.56 ± 0.52	−0.41 ± 0.59
Reflection index, au	0.28 ± 0.14	0.18 ± 0.08	0.21 ± 0.10	0.27 ± 0.11	0.31 ± 0.11	0.36 ± 0.12	0.41 ± 0.13
Stiffness index, m/s	7.8 ± 2.4	6.2 ± 1.0	6.7 ± 1.1	7.5 ± 1.0	8.1 ± 1.6	8.9 ± 2.8	10.3 ± 3.4
*Vascular beds*							
Systemic vascular resistance, 10^6^ Pa·s·m^−3^	173.7 ± 42.5	153.8 ± 34.5	159.5 ± 36.5	171.2 ± 38.3	178.9 ± 41.0	188.6 ± 43.8	198.1 ± 45.1
Peripheral vascular compliance, 10^9^ m^3^/Pa	29.3 ± 7.7	40.1 ± 0.0	35.5 ± 0.0	31.0 ± 0.0	26.4 ± 0.0	21.9 ± 0.0	17.3 ± 0.0
Time constant, s	1.07 ± 0.39	1.30 ± 0.41	1.22 ± 0.42	1.12 ± 0.36	1.02 ± 0.32	0.90 ± 0.28	0.82 ± 0.26

Values are means ± SD; *n*, no. of subjects. See glossary for definition of terms.

### Comparison with In Vivo Data

**Fig. 6.**
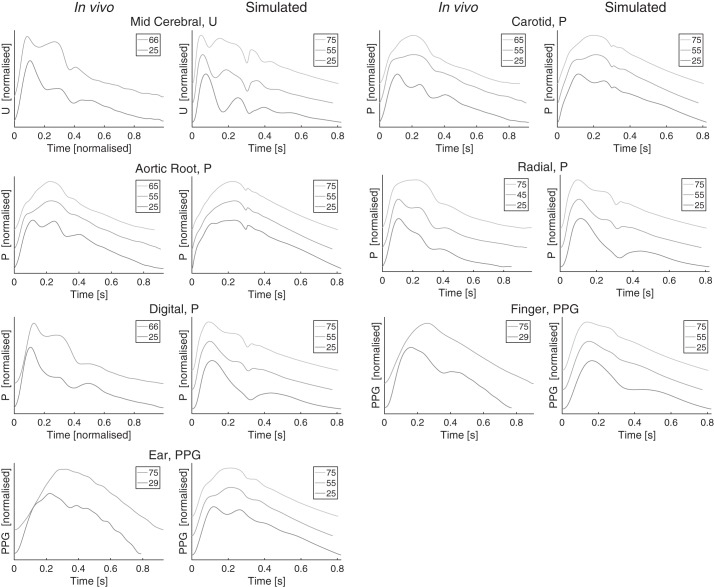
A comparison between simulated and in vivo pulse wave (PW) shapes. Each pair of plots shows in vivo PWs on the *left*, and simulated PWs on the *right*. PWs are shown for different ages in each plot, offset and normalized. Legends indicate ages. In vivo data were obtained from Flück et al. [(46; licensed under CC-BY 3.0; https://creativecommons.org/licenses/by/3.0/.] normotensive patients undergoing screening for hypertension [described in Li et al. (89)], and the Vortal data set [described in Charlton et al. (28, 29)]. See glossary for definition of terms.

A selection of the simulated PWs are compared with PWs from the literature in Fig. 6. PWs from both the PW database (simulated) and the literature (in vivo) are shown for young, middle-aged, and elderly subjects. The shapes of the simulated PWs changed with age in a similar manner to the in vivo PWs: *1*) the amplitude of the secondary systolic peak in middle cerebral *U* PWs increased with age; *2*) the augmentation in the secondary systolic peak of the carotid and ascending aorta pressure PWs increased with age; *3*) the diastolic peak in the radial, digital, and femoral (not shown) pressure PWs was present for the 25 yr old and disappeared with age; *4*) the diastolic peak of the finger PPG PW disappeared with age; *5*) the two systolic peaks in the ear PPG merged with age.

**Fig. 7.**
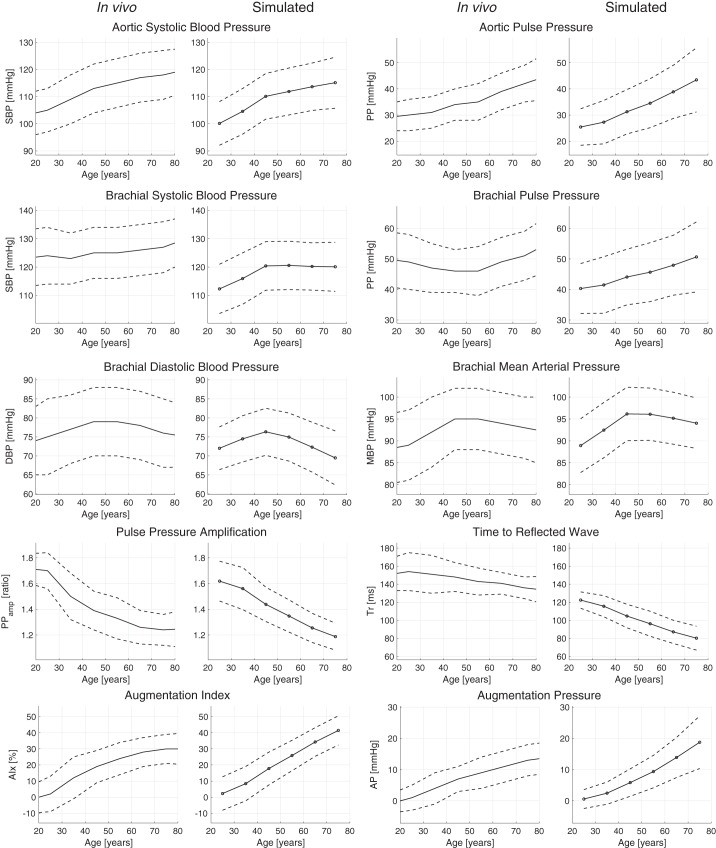
A comparison between in vivo hemodynamic characteristics (*left-hand* plots) and the characteristics of the simulated pulse wave data set (*right-hand* plots). Solid lines indicate mean values, and dashed lines indicate ±1 SD. In vivo data are from McEniery et al. (99). See glossary for definition of terms.

The hemodynamic characteristics of the simulated PWs are compared with those in the literature in Fig. 7. The changes with age were mostly similar between the literature (*left-hand* plots) and simulated (*right-hand* plots) characteristics: aortic pressure, systolic pressure, and PPs increased with age; PP_amp_ decreased with age; the time to the return of the reflected pressure wave (Tr) decreased with age; and pressure augmentation increased with age (AIx and AP). However, brachial PP increased with age, rather than decreasing and then increasing with age. This was because the brachial SBP was slightly lower than in the literature at ages 25 and 35 yr. Overall, these similarities indicate that the hemodynamic characteristics of the simulated PWs showed similar trends, and in most cases similar absolute values, to those reported in the literature.

### Case Studies

#### The determinants of changes in PP_amp_ with age.

**Fig. 8.**
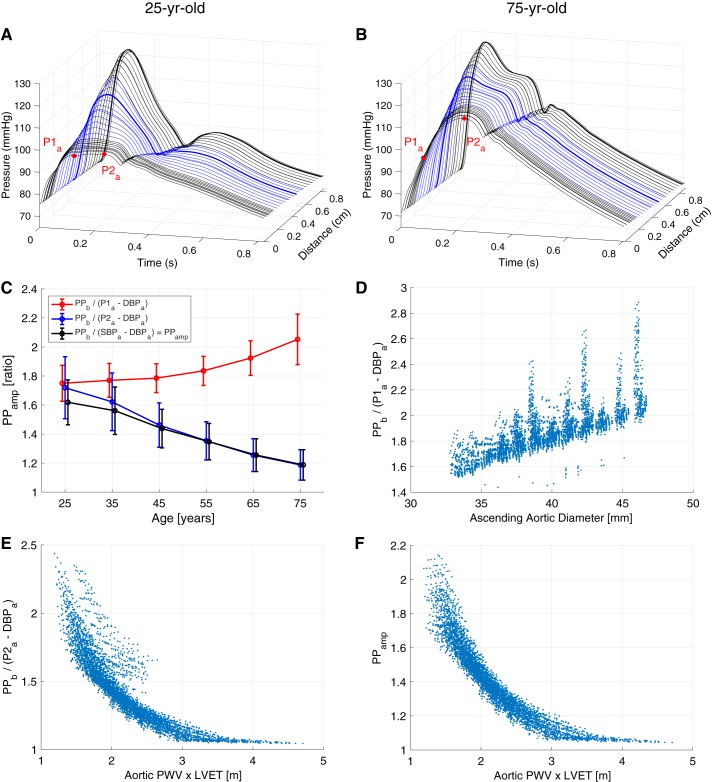
The causes of changes in pulse pressure (PP) amplification (PP_amp_) with age. *A* and *B*: how the pressure pulse wave (PW) changed with distance along the path from the aortic root to the finger for young and elderly baseline subjects (blue indicates PWs in the subclavian and brachial arteries). *C*: PP_amp_ values (mean ± SD) calculated using aortic diastolic (DBP) and systolic blood pressure (SBP; black), early systolic pressure (P1_a_; red), and late systolic pressure (P2_a_; blue). *D*–*F*: the principal cardiovascular determinants of early systolic amplification, late systolic augmentation, and PP_amp_, respectively. Subscripts “a” and “b” indicate aortic and brachial, respectively; LVET, left ventricular ejection time; PWV, pulse wave velocity.

The profiles of pressure PW propagation from the aorta to the brachial artery were examined in young and elderly subjects, as shown in Fig. 8, *A* and *B*. The profiles demonstrate that two mechanisms influence PP_amp_ (PP_amp_ = PP_b_/PP_a_; subscripts “a” and “b” indicate aortic and brachial, respectively). First, the early systolic portion was amplified in both subjects, causing SBP_b_ to be greater than SBP_a_ and therefore PP_amp_ > 1. Second, late systolic aortic pressure augmentation (the increase in pressure from P1_a_ to P2_a_) was higher in older subjects, increasing PP_a_ and decreasing PP_amp_. The contributions of these mechanisms to PP_amp_ for the whole database are illustrated in Fig. 8*C*. The amplification of the early systolic portion increased with age, as shown in red by PP_b_/(P1_a_ − DBP_a_). In contrast, the increase in late systolic aortic pressure augmentation with age (in blue) caused a decrease in PP_b_/(P2_a_ − DBP_a_) with age. The effect of aortic pressure augmentation outweighed that of early systolic amplification, meaning PP_amp_ decreased substantially with age, in keeping with in vivo studies (Fig. 7). The database can be used to gain insight into the CV determinants of these mechanisms: early systolic amplification was determined primarily by the diameter of the larger arteries, and late systolic aortic pressure augmentation was largely determined by PWV and LVET, as shown in Fig. 8, *D* and *E*. Indeed, since PP_amp_ was primarily determined by late systolic aortic pressure augmentation, it was largely determined by arterial stiffness (i.e., PWV) and LVET, as shown in Fig. 8*F*. The change in PP_amp_ observed with age was primarily due to changes in aortic pressure wave morphology.

#### Noninvasive peripheral assessment of aortic stiffness.

**Fig. 9.**
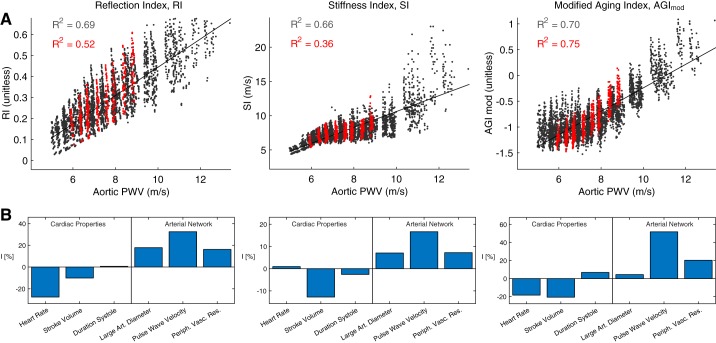
The correlation of photoplethysmogram (PPG)-derived pulse wave (PW) indexes with aortic pulse wave velocity (PWV; *A*), and their physiological determinants (*B*). Data derived for all virtual subjects are shown in black, whereas red indicates data from 45-yr-old subjects. AGI_mod_, modified aging index; I, relative sensitivity index; RI, reflection index; SI, stiffness index.

The performance of the PPG-derived indexes for assessing aortic stiffness is shown in Fig. 9. All three correlated with aortic PWV, with similar coefficient of determination (*R*^2^) values ranging from 0.66 to 0.70 (Fig. 9*A*). This indicates that these indexes may have utility for assessing aortic stiffness, in line with findings of clinical studies. However, the *R*^2^ values for the RI and SI were lower when using only data from middle-aged (45 yr old) virtual subjects (shown in red), indicating that these indexes may be less useful for stratifying middle-aged patients. The sensitivity analyses in the lower plots quantify the relative impact of different input parameters on the indexes. Several CV properties in addition to PWV influenced the indexes, such as HR and SV. For instance, the RI and SI both increased with large-artery diameter. Since large-artery diameter and aortic PWV both increase with age, this strengthened their correlations with aortic PWV across the age range. In contrast, the AGI_mod_ was not strongly influenced by large-artery diameter and performed better both across the age range and when considering only middle-aged subjects. This in silico assessment of PPG-derived indexes for assessing aortic stiffness indicates that *1*) clinical studies should investigate performance over a small age range as well as over the entire cohort to assess the potential utility of indexes for stratifying patients; *2*) the AGI_mod_ may provide best performance for stratification of middle-aged patients; and *3*) indexes can also be influenced by HR and SV, indicating that it may be beneficial to assess performance when these CV properties are varied in vivo.

#### Cardiac output monitoring.

**Fig. 10.**
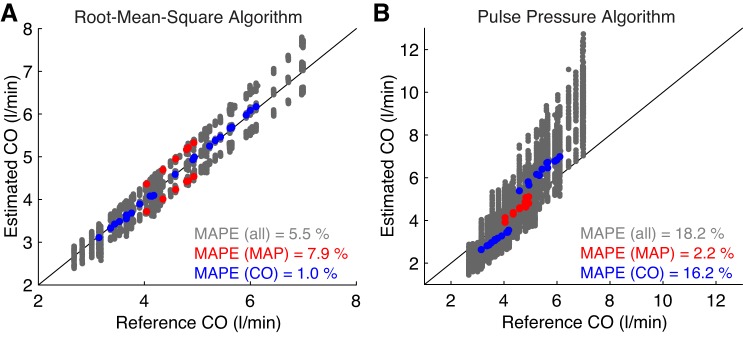
Estimated versus reference cardiac output (CO) for root-mean-square (*A*) and pulse pressure (*B*) CO algorithms. Data in red and blue correspond to simulations in which either mean arterial pressure (MAP) or CO, respectively, were changed from baseline while all other parameters were held constant. MAPE, mean absolute percentage error.

The performance of the CO algorithms is shown in Fig. 10. Overall, the RMS algorithm performed better with a MAPE of 5.5% compared with 18.2% for the PP algorithm. However, a subgroup analysis of performance during changes in MAP and CO revealed that the algorithms had different strengths and weaknesses. The PP algorithm performed better during changes in MAP (MAPE of 2.2% compared with 7.9%), whereas the RMS algorithm performed better during changes in CO (MAPE of 1.0% compared with 16.2%). Therefore, different algorithms may be more appropriate for different clinical settings. For instance, in the critical care setting, CO algorithms should ideally remain accurate during administration of vasoactive drugs, which can affect MAP (104). Furthermore, clinical studies should assess the performance of CO algorithms during changes in those CV properties that would be expected to change in clinical use. Had this study only considered changes in CO, and not MAP, then the potential weakness of the RMS algorithm would not have been identified.

## DISCUSSION

In this study, we developed and verified an approach for simulating PWs representative of a sample of healthy adults. 1D numerical modeling was used to simulate PWs for virtual subjects of different ages, where the input parameters were based on normal values and ranges of CV properties obtained from a comprehensive review of previous studies. The simulated PWs exhibited similar changes with age to those reported in in vivo studies, including changes in PW shape and in hemodynamic parameters derived from PWs. The utility of this approach for gaining novel insights into hemodynamics and PW indexes was demonstrated through three case studies. The approach for simulating PWs, the resulting PW database, and the accompanying code are valuable resources for future in silico studies of hemodynamics and PW indexes.

### Approach for Simulating PWs

We used 1D modeling combined with a comprehensive review of CV changes with age to simulate PWs around the body for healthy subjects of different ages. The use of 1D modeling allowed us to simulate PWs at a range of common measurement sites, similar to previous studies (107, 169), incorporating the effects on PW propagation of changing arterial properties through the arterial tree. The model input parameters were adjusted to simulate PWs for different ages. The input parameters were based on a literature review, which identified normal values and ranges of the parameters, building on previous reviews (17, 47, 77, 83–85, 98, 109, 112, 134). Parameters were changed with age, allowing the effects of aging to be investigated, and were also varied within normal ranges at each age, allowing the influences of individual parameters to be elucidated. This builds on previous work modeling changes with age (34, 39, 59, 60, 94, 114, 119).

Particular strengths to this approach are as follows. First, it incorporates relationships between some input parameters, including the dependencies of LVET on SV and HR, and arterial stiffness on MAP and arterial geometry. Second, it simulates the PPG, which is of particular interest, given the widespread use of PPG sensors in smart watches and fitness bands. We simulated the PPG from the blood volume in terminal windkessel models because pulsatile blood volume is commonly cited as the main determinant of the PPG (4). Other approaches that have previously been used to simulate the PPG in 1D modeling include assuming the PPG is proportional to *A* (44) and using a transfer function to estimate the PPG from P (30). This methodology for simulating the PPG needs further investigation to understand whether it is truly representative of PPG PWs measured in vivo.

The approach was verified by comparing changes in simulated PWs with age to those observed in vivo. The main finding, that simulated PWs exhibited similar changes to those observed in vivo, provides confidence that the approach produces realistic changes with age. This is complementary to previous studies that used 1D modeling to simulate PWs at different ages (59, 61, 114).

The main limitations to the approach are as follows. First, the literature review included mostly cross-sectional rather than longitudinal studies. Consequently, the differences in simulated PWs with age can be expected to be representative of those that would be observed between subjects of different ages, rather than those that occur within an individual over time. Second, we found only minimal evidence in the literature describing how some CV properties change with age, namely, PVC and the diameters of more peripheral arteries. Third, insufficient evidence was found to model the associations between certain parameters. For instance, the subjects with abnormally high PP (described in *Database Characteristics* above) mostly had combinations of CV properties that would be expected to produce high PP; e.g., due to increased SV and/or decreased arterial compliance (35). It would be helpful to incorporate further information on correlations between parameters, such as those that influence PP, when it becomes available in the literature: doing so may reduce the number of subjects exhibiting BPs outside healthy ranges. Fourthly, the approach does not incorporate methodology for adjusting the arterial network geometry in line with variation in height and body surface area, an important consideration when investigating sex-associated differences in hemodynamics (133). This may be a valuable extension in the future, as it would allow for investigation of the influence of network geometry on hemodynamics, such as the influence of height on aortic pressure augmentation (11, 70, 71) and PP (87). Indeed, incorporating sex-specific CV properties could provide valuable insight into the determinants of differences in PW features between females and males (99). Fifth, the PW database is designed to be representative of healthy adults: it may be helpful to adapt it to study PWs in diseases, such as hypertension and peripheral arterial disease. It should also be noted that PPG PWs can only be measured at peripheral locations (such as the finger, wrist, and arm). Consequently, simulated PPG PWs at central locations (such as the aorta) are currently not of practical significance.

### Application

The utility of the approach for simulating PWs was demonstrated through case studies, which present interesting findings in keeping with in vivo studies and indicate directions for future research.

The first case study provided insight into the mechanisms underlying changes in PP_amp_ with age. PP_amp_ has previously been proposed as an indicator of CV risk suitable for use in population studies (14). If it is to be used for this purpose, then it is important to have a thorough understanding of the mechanisms behind it. The first mechanism identified in this study, the increased contribution of late systolic aortic pressure augmentation with age, has also been observed in in vivo studies (8, 122, 143, 168). In this case study, the controlled changes in CV properties in the database were used to identify the determinants of late systolic aortic pressure augmentation: arterial stiffness and cardiac ejection properties, as observed previously (52, 161). The second mechanism, the contribution of early systolic pressure amplification, has been less well reported. A nonsignificant trend of increased early systolic pressure amplification with age was reported in Wilkinson et al. (167). This case study adds evidence to support this finding and indicates that this mechanism may be more pronounced in subjects aged 75 yr and older.

The second and third case studies investigated the performance of PW indexes for assessing aortic stiffness and CO. This approach of assessing PW indexes in silico could inform the design of future clinical studies. In both case studies, the PW indexes were found to be influenced by other CV properties besides those they aimed to assess. PPG-derived indexes for assessing aortic stiffness were determined in part by cardiac properties (SV and HR), whereas the accuracy of BP-derived indexes for tracking changes in CO was influenced by MAP and CO itself. These findings indicate that future studies of these indexes should assess their performance during changes in these properties. In addition, the performance of some indexes for assessing aortic stiffness was reduced when only considering subjects of a certain age. Whereas previous in vivo studies have provided valuable results across a wide age range (104, 164), this study highlights the importance of also assessing indexes across a small age range to assess their utility for risk stratification.

### Perspectives

The approach presented for simulating PWs may be useful for obtaining insight into the hemodynamic mechanisms underlying findings of previous in vivo studies, and for designing novel in vivo studies. Similar approaches have previously been used to identify the mechanisms underlying in vivo observations, including *1*) the reasons for differences in the performance of different PWV measurement paths for assessing aortic PWV (169); *2*) the CV properties that influence a transfer function relating peripheral to central pressure (74, 150); and *3*) the strengths and weaknesses of physiological measurement devices (115, 156). More recently, studies have used both in vivo PW measurements and simulated PWs to obtain novel insights into hemodynamics, including *1*) the determinants of central PP (161); and *2*) the influence of CV properties on forward and backward pressure waveform morphology (89). We expect that the approach presented here, which has been verified against in vivo data, will be of value for future studies.

In the future, this approach may form a basis for creating hemodynamic digital twins: simulations of an individual’s hemodynamics using input parameters obtained from their physiological measurements (155). This would allow changes in CV health to be identified when an individual’s PWs, acquired by smart wearables, diverge from their digital twin’s “normal” PWs, prompting clinical assessment.

This article is accompanied by resources to enable other researchers to use this approach for simulating PWs. First, the PW database is freely available to download (32). Second, key fiducial points on PWs (such as those labeled in Fig. 4) are provided, allowing researchers to use the results of PW analysis without performing any signal processing. Third, the code used to create and analyze the PW database and for reproducing the case studies is available, allowing researchers to run example analyses and gain an understanding of how to use the database (33). Fourth, the signal processing tool used to extract PW indexes, PulseAnalyse, is available (26). It is currently designed for use with this database, and work is ongoing to develop it on independent data sets. Further details of these resources are provided in the endnote at the end of this article.

### Conclusion

We have designed and verified an approach for simulating PWs representative of healthy adults of different ages. A computational model of the arterial system was used to simulate several types of PWs at common measurement sites for 4,374 virtual subjects. Simulations were performed for subjects of different ages by adjusting model input parameters in line with typical CV parameters for each age obtained from a comprehensive literature review. The resulting database of PWs exhibited similar age-related changes in hemodynamic parameters and PW morphology to those in previous in vivo studies. We demonstrated the utility of the approach through case studies, which provided novel insights into the hemodynamic determinants of PWs and provided pilot data to inform clinical studies of PW algorithms. The database is freely available and is a valuable resource for future research.

## GRANTS

This work was supported by the British Heart Foundation [PG/15/104/31913], the Wellcome Engineering and Physical Sciences Research Council (EPSRC) Centre for Medical Engineering at King’s College London [WT 203148/Z/16/Z], and the King’s College London and Imperial College London EPSRC Centre for Doctoral Training in Medical Imaging [EP/L015226/1] funding for J.M.H. The authors acknowledge financial support from the Department of Health through the National Institute for Health Research (NIHR) Cardiovascular MedTech Co-operative at Guy’s and St. Thomas’ National Health Service Foundation Trust (GSTT).

## DISCLAIMERS

The views expressed are those of the authors and not necessarily those of the British Heart Foundation, Wellcome Trust, EPSRC, NIHR, or GSTT.

## DISCLOSURES

No conflicts of interest, financial or otherwise, are declared by the author(s).

## AUTHOR CONTRIBUTIONS

P.H.C., J.M.H., S.V., Y.L., P.C., and J.A. conceived and designed research; P.H.C. and J.A. performed experiments; P.H.C. analyzed data; P.H.C., S.V., Y.L., P.C., and J.A. interpreted results of experiments; P.H.C. and J.A. prepared figures; P.H.C., P.C., and J.A. drafted manuscript; P.H.C., J.M.H., S.V., Y.L., P.C., and J.A. edited and revised manuscript; P.H.C., J.M.H., S.V., Y.L., P.C., and J.A. approved final version of manuscript.

## ENDNOTE

At the request of the authors, readers are herein alerted to the fact that additional materials related to this manuscript may be found at https://doi.org/10.5281/zenodo.3374476. These materials are not a part of this manuscript, and have not undergone peer review by the American Physiological Society (APS). APS and the journal editors take no responsibility for these materials, for the website address, or for any links to or from it.

## Figures and Tables

**Fig. A1.**
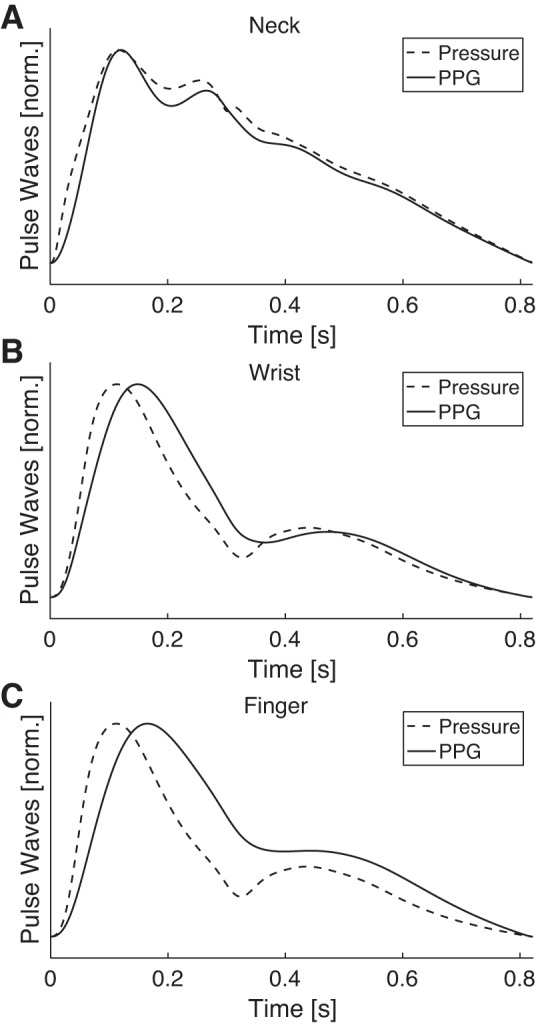
Exemplary simulated photoplethysmogram (PPG) pulse waves (solid lines) compared with the corresponding pressure pulse waves at three sites: carotid artery (neck; *A*); radial artery (wrist; *B*); and digital artery (finger; *C*). Pulse waves have been normalized to occupy the same range. Data were taken from the 25-yr-old baseline subject.

**Fig. A2.**
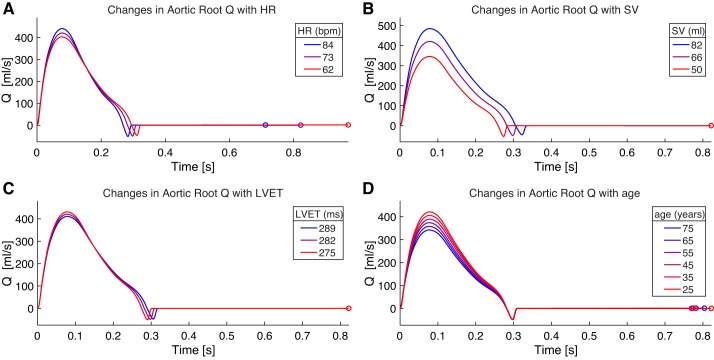
Simulated aortic flow waves obtained for independent changes in inflow characteristics from the 25-yr-old baseline subject and obtained for baseline subjects of different ages. *A*–*C*: flow waves obtained by varying heart rate (HR), stroke volume (SV), and left ventricular ejection time (LVET), respectively, by ±1 SD from the mean value for the 25-yr-old baseline subject while all other input parameters are held at baseline values. *D*: flow waves obtained for the baseline subjects of different ages. bpm, Beats/min; Q, flow rate.

## APPENDIX

### Numerical Model

#### Arterial network geometry.

The geometry of the baseline 25-yr-old model is provided in the supplementary file (see endnote.) The following information is provided for each of the 116 arterial segments in the baseline model: length, inlet and outlet radii, and inlet and outlet nodes. The geometry for each of the virtual subjects is provided in the PW database.

The geometry was adapted from the arterial network presented in Mynard and Smolich (107), by taking the following steps (which are documented in the supplemental file):

• *Segments 1*, *2*, and *3* (107), which represent the left ventricular outflow tract, aortic root, and ascending aorta, were combined into a single segment (*segment 1* in the new network).
• *Segments 10*, *12*, and *14* (107), which represent the latter part of the right subclavian artery, the right axillary artery, and the right brachial artery, were combined into a single segment (*segment 7* in the new network).
• An additional segment (*segment 30* in the new network) was added, extending the celiac artery by 10 mm.
• *Segments 81*, *84*, *85*, *86*, *91*, *92*, *102*, *121*, and *123* in (107), representing the basilar artery, the initial parts of the posterior cerebral arteries, the distal internal carotid arteries, and anterior communicating artery, were adjusted (mainly by adjusting their lengths).
• The luminal areas of each segment obtained from (107) were increased by a scaling factor of 1.5 to increase the compliance of the network and reduce the simulated PPs, making them more similar to those reported in (107).
• We added arterial *segments 97–116* in our network to represent the larger arteries of the hand. These were adapted from Alastruey et al. (3) using the *calculate_hand_artery_segment_radii.m* script (see endnote for access). Briefly, the areas of the distal segments at the junctions at the end of the radial and ulnar arteries were adjusted to achieve *A* ratios of 1.15 as suggested for matched conditions in Greenwald and Newman (58). The remaining luminal areas of the hand were adjusted from their original values, in line with the adjustments made to achieve matched junctions.

#### Simulating the PPG.

The methodology used to simulate PPG PWs was introduced in *Modeling Arterial Pulse Waves* above. We now provide additional details of the methodology used in the two possible scenarios: *1*) at the periphery (i.e., the end of a 1D model terminal branch); and *2*) within the arterial network. At the periphery (such as the digital artery in the finger), the PPG was calculated using

(A1) $$\text{PPG}\left(t\right)=\overset{t}{\underset{0}{\int }}Q_{1\text{D}}\left(t^{\prime }\right)-Q_{\text{out}}\left(t^{\prime }\right)\text{d}t^{\prime }$$

where *Q*_1D_ is the inflow to the terminal windkessel, and *Q*_out_ is the outflow (as shown in Fig. 1). At distal sites within the arterial network (such as the wrist), the PPG was calculated by assuming that the volume of blood in the microvasculature at that site could be modeled by a windkessel model. The basis for this assumption is that vascular beds at sites within the arterial network are perfused by arterioles branching from the major artery at that site (e.g., the radial artery at the wrist), which are too small to be represented in the arterial network. Therefore, the inflow to the windkessel was assumed to be proportional to the flow through the arterial segment, at a pressure equal to that of the arterial segment. The same equation was used to calculate the PPG, where *Q*_1D_ was set equal to the flow through the arterial segment, and *Q*_out_ was calculated using

(A2) $$Q_{\text{out}}\left(t\right)=\frac{\text{P}\left(t\right)-{\text{P}}_{\text{out}}}{R}$$

where

(A3) $$R=\frac{\bar{\text{P}\left(t\right)}-{\text{P}}_{\text{out}}}{\bar{Q_{1\text{D}}\left(t\right)}}$$

and P_out_ is the outflow pressure (with P and *Q*_1D_ obtained at the point of measurement). This approach was verified by checking that a PPG PW calculated using this approach at the periphery is very similar to the one calculated using the flow in and out of the terminal windkessel. Figure A1 shows examples of the resulting PPG PWs at common measurement sites.

### Literature Review

Table A1 presents the results of the literature review for each model input parameter. Table A2 provides equations for each input parameter and its SD, which were calculated using data from articles selected from the literature review presented in *Prescribing Model Input Parameters for Different Ages Based on a Literature Review* above.

### Prescribing Model Parameters

#### The aortic inflow waveform.

Each virtual subject’s aortic inflow waveform was calculated from the template waveform to achieve the desired inflow characteristics (HR, SV, LVET, PFT, and RFV). This was performed using the *AorticFlowWave* script (see the endnote for access), which ensures that the morphology of each segment of the inflow wave (systolic upslope, systolic downslope, and reverse flow) remains the same during changes in inflow wave characteristics. Figure A2 shows the simulated aortic flow waves obtained for independent changes in inflow characteristics from the 25-yr-old baseline subject and obtained for baseline subjects of different ages. Note that the values for LVET change when varying HR and SV, in accordance with the relationship between LVET and HR and SV given by *Eq. 1*. These changes in LVET solely affect the diastolic downslope portion of the flow wave, ensuring that PFT remains constant during these changes.

#### Arterial stiffness.

The relationship between arterial stiffness and radius given by *Eq. 2* was adjusted for each virtual subject to minimize the differences between the desired PWVs and the expected PWVs along three paths: carotid-femoral, brachial-radial, and femoral-ankle. This was performed using the *calculate_pwdb_input_parameters.m* script (see endnote for access). The values for the constants (*k*_1_, *k*_2_, and *k*_3_) in *Eq. 2* were obtained as follows. *k*_1_, which determines the stiffness of smaller arteries, was set to 3 × 10^6^ g·s^−2^·cm^−1^, following Mynard and Smolich (107). The value for *k*_2_, which determines the point of transition in stiffness between larger and smaller arteries, was adjusted slightly from the value of −9 cm^−1^ used in Mynard and Smolich (107) to −13.5 cm^−1^, as this was found to give more realistic PW shapes and PP_amp_. The value for *k*_3_, which determines the stiffness of larger arteries, was optimized for each virtual subject by minimizing the absolute difference between the desired and expected carotid-femoral PWV. The desired values were influenced by age and normal variation in MAP and PWV. For the baseline subject at each age (with age-specific baseline values for MAP and PWV), *k*_3_ ≈ 430,118 – 1871.3 × age + 244.11 × age^2^ g·s^−2^·cm^−1^.

### Pulse Wave Analysis Algorithms

PW analysis was performed using the *PulseAnalyse* script (see the endnote for access). The methods used for detecting each of the fiducial points (see Fig. 4) on the pressure and PPG PWs are now described.

PWs were preprocessed by *1*) removing very high frequencies with a low-pass filter with −3-dB cutoff frequency of 16.75 Hz; *2*) removing very low frequencies by subtracting any linear trend between PW onset and end; and *3*) aligning PWs to start at the beginning of the systolic upslope. First, second, and third derivatives were calculated using a first-derivative Savitzky-Golay filter with a window size of five samples (140). The fourth derivative was calculated from the third derivative using a first-derivative Savitzky-Golay filter with a window size of nine samples.

Fiducial points were then identified using the criteria listed in Table A3. These criteria are adapted from Charlton et al. (30). PW indexes were calculated from these fiducial points as described in Charlton et al. (30). The AIx and AP were calculated using *p1in* and *p2pk* (referred to as P1 and P2 in Fig. 4). The stiffness index was calculated by assuming a height of 1.75 m, in keeping with Mynard and Smolich (107).

**Table A1.** A summary of the literature review of changes in cardiovascular properties with age

			Change with Age, %				Data Source, Ref. No.	
Cardiovascular Property	No. Studies	No. Articles	None	Increase	Decrease	Nonlinear	Change	Variation
*Cardiac*								
Heart rate	22	22	86.4	4.5	4.5	4.5	173	118
Stroke volume	11	11	18.2	9.1	72.7	0.0	120	120
Left ventricular ejection time	10	10	80.0	10.0	0.0	10.0	107	55
Peak flow time	3	3	66.7	0.0	33.3	0.0	73	73
Reverse flow volume	1	1	100.0	0.0	0.0	0.0	15	15
*Arterial*								
Length								
Proximal aorta	5	4	0.0	100.0	0.0	0.0	66	15
Distal aorta	5	4	60.0	20.0	0.0	20.0		
Carotid	1	1	100.0	0.0	0.0	0.0		
Iliac	1	1	100.0	0.0	0.0	0.0		
Diameter								
Ascending aorta	13	13	7.7	92.3	0.0	0.0	66	1
Descending thoracic aorta	5	5	0.0	100.0	0.0	0.0	66	1
Abdominal aorta	6	6	0.0	100.0	0.0	0.0	66	1
Carotid	6	6	33.3	66.7	0.0	0.0	63	63
Iliac	2	2	50.0	50.0	0.0	0.0		
Femoral	3	3	66.7	33.3	0.0	0.0		
Brachial	2	2	0.0	100.0	0.0	0.0		
Radial	1	1	0.0	100.0	0.0	0.0		
Pulse wave velocity								
Aorta	24	19	4.2	95.8	0.0	0.0	127a	127a
Upper limb	11	11	0.0	100.0	0.0	0.0	9	
Lower limb	5	5	20.0	80.0	0.0	0.0	9	
*Vascular beds*								
Systemic vascular resistance	9	9	44.4	55.6	0.0	0.0	99	99
Systemic vascular compliance	5	5	0.0	0.0	100.0	0.0	100	129

The type of change with age used for each parameter is underlined, and references to the relevant articles are provided in the last columns.

**Table A2.** The model input parameters, where the mean and standard deviation can vary with age (in yr)

Cardiovascular Property	Mean Value	Standard Deviation
*Cardiac*		
Heart rate, beats/min	Nonlinear, see text	11.2
Stroke volume, ml	72.7–0.253 × age	18.1–0.081 × age
Left ventricular ejection time, ms	282	23.3
Peak flow time, ms	79.0	11.0
Reverse flow volume, ml	0.730	0.630
*Arterial*		
Length of proximal aorta, % of 25-yr-old	80.0 + 0.800 × age	10.7 + 0.107 × age
Diameter of larger arteries, % of 25-yr-old	90.9 + 0.365 × age	8.18 + 0.033 × age
Pulse wave velocity	Nonlinear, see text	Nonlinear, see text
*Vascular beds*		
Mean arterial blood pressure, mmHg	Nonlinear, see text	7.98–0.00952 × age
Peripheral vascular compliance, % of 25-yr-old	128.4–1.136 × age	35.2–0.311 × age

Coefficients are given to three significant figures. % of 25-yr-old indicates the percent change from the value(s) in the 25-yr-old baseline model.

**Table A3.** The criteria used to identify fiducial points on the pressure and photoplethysmogram pulse waves

Fiducial Point	Criterion for Finding Location
*s*, Systolic peak	Maximum of *x*
*ms*, Maximum slope	Maximum of *x′*
*a*	The highest local maximum of *x″* between an initial buffer of 0.005 seconds and *ms*. If no local maximum is found in this region, then *a* is defined as the last local maximum before the initial buffer.
*b*	The lowest local minimum of *x″* between *a* and an upper bound of 25% of the PW duration.
*p1in*	Two candidate locations for *p1in* are identified as *1*) the first local minimum on *x′* after 0.1 s; and *2*) the second local minimum (if it exists, otherwise the first) on *x′* after *b*. *p1in* is taken to be the candidate location that occurs first. If this is later than 0.18 s, then *p1in* is updated to be the first local minimum in *x″″* after 0.1 s. If *p1in* is still later than 0.18 s, then it is updated to be the last local minimum in the first derivative before 0.18 s.
*e*	A candidate location for *e* is identified as the highest local maximum on *x″* between *ms* and 60% of the PW duration. If this is the first local maximum within this search region, then it may be the *c* point. To check for this, inflection points are identified between *b* and this candidate location (from local minima on *x‴*). If there are no inflection points, and if there is one local maximum in this search region, then update the candidate location to be the first local maximum on *x″* at or after 60% of the PW duration.
*c*	*c* is identified as the highest local maximum on *x″* between *b* and *e*. If there are no local maxima in this search region, then identify *c* as the lowest local minimum on *x‴* after *b* and before *e*.
*dic*, Dicrotic notch	*dic* is coincident with *e.*
*dia*, Diastolic peak	If there is one or more local maxima on *x* after *dic* and before 80% of the PW duration, then take the first local maximum as *dia*. If there is not, then take the first local maximum on *x′* after *e* and before 80% of the PW duration.
*d*	*d* is identified as the lowest local minimum on *x″* between *c* and *e*, unless there is not a local minimum in this search region, in which case take *d* as coincident with *c*.
*p2in*	A candidate location for *p2in* is taken as the last local minimum on *x‴* before *d*. If this location is before *p1in*, then it is updated to be the last local minimum on *x‴* before *e*. If there is one or more local maxima on *x* between the candidate location and *e*, then take the last local maximum as *p2in*.
*p1pk* and *p2pk*	Initial locations of *p1pk* and *p2pk* are set to the locations of *p1in* and *p2in*. Either *p1pk* or *p2pk* is adjusted to be coincident with *sys* (determined by whichever of *p1in* or *p2in* is closest to *sys*). Each of *p1pk* and *p2pk* is then adjusted to be at a nearby local maximum on *x*, if there is a local maximum that satisfies the following criteria. The maximum must lie between the mean of the candidate locations of *p1pk* and *p2pk*, and *ms* for *p1pk*, and *e* for *p2pk*. It must also be higher than the candidate locations. If more than one maximum satisfies these criteria, then the maximum with the highest value is taken.

*x*, Pulse wave (PW); *x′*, first derivative of PW; *x″*, second derivative of PW; *x‴*, third derivative of PW; *x″″*, fourth derivative of PW.

## GLOSSARY

### Abbreviations

Γ: Arterial wall viscosity
μ: Blood viscosity
ρ: Blood density
1D: One-dimensional
*A*: Area
AGI_mod_: Modified aging index
AIx: Augmentation index
AP: Augmentation pressure
au: Arbitrary units
BP: Blood pressure
bpm: Beats/min
*c*_d_: Diastolic wave speed
CO: Cardiac output
CT: Crest time
CV: Cardiovascular
DBP: Diastolic blood pressure
Dia: Diameter
dia: Diastolic peak
dic: Dicrotic notch
*E*: Young’s modulus
h: Arterial wall thickness
HR: Heart rate
I: Relative sensitivity index
LVET: Left ventricular ejection time
MAP: Mean arterial pressure
MAPE: Mean absolute percentage error
MRI: Magnetic resonance imaging
*ms*: Point of maximal slope
P: Pressure
P1: Pressure at first shoulder
P2: Pressure at second pressure peak
PFT: Peak flow time
PP: Pulse pressure
PP_amp_: Pulse pressure amplification
PPG: Photoplethysmogram
PTT: Pulse transit time
PVC: Peripheral vascular compliance
PVR: Peripheral vascular resistance
PW: Pulse wave
PWV: Pulse wave velocity
*Q*: Flow rate
*R*^2^: Coefficient of determination
*R*_d_: Diastolic arterial radius
RFV: Reverse flow volume
RI: Reflection index
RMS: Root mean square
s: Systolic peak
SBP: Systolic blood pressure
SD: Standard deviation
SI: Stiffness index
SV: Stroke volume
SVR: Systemic vascular resistance
*T*: Cardiac period
*t*_dia_: duration of diastole
Tr: Time to return of the reflected pressure wave
*U*: Flow velocity
